# Surgical Recommendations for Black Women Compared to White Women With Ovarian Cancer in the US

**DOI:** 10.7759/cureus.108834

**Published:** 2026-05-14

**Authors:** Oluwasegun Akinyemi, Mojisola Fasokun, Akachukwu N Eze, Fadeke Ogunyankin, Chibuzo C Eze, Temitope Ogundare, Ofure Omokhodion, Seun Ikugbayigbe, Gabriella Kuffour, Kakra Hughes, Guoyang Luo, Shari Lawson

**Affiliations:** 1 Department of Obstetrics and Gynecology, The Clive O Callender Outcomes Research Center, Howard University College of Medicine, Washington DC, USA; 2 Department of Epidemiology, University of Alabama at Birmingham, Birmingham, USA; 3 College of Medicine, The Clive O Callender Outcomes Research Center, Howard University College of Medicine, Washington DC, USA; 4 Department of Clinical and Translational Science, Cook Children's Health Care System, Fort Worth, USA; 5 Department of Medicine, Emory University School of Medicine, Atlanta, USA; 6 Department of Psychiatry, Boston University School of Medicine, Boston, USA; 7 Department of Family Medicine, University of Iowa Hospitals and Clinics, Iowa City, USA; 8 Department of Biological Sciences, Eastern Illinois University, Charleston, USA; 9 Department of Obstetrics and Gynecology, University of Central Florida, Orlando, USA; 10 Department of Surgery, Howard University Hospital, Washington DC, USA; 11 Department of Obstetrics and Gynecology, Inova Health System, Fairfax, USA; 12 Department of Obstetrics and Gynecology, Howard University College of Medicine, Washington DC, USA

**Keywords:** cancer treatment, health equity, inverse probability weighting, multiple imputation, ovarian cancer, race, seer, surgical disparities

## Abstract

Objective: To evaluate racial disparities in surgical treatment recommendations for Black and White women diagnosed with ovarian cancer in the United States.

Methods: We conducted a retrospective cohort study using SEER data from 2001 to 2021. The primary outcome was a recommendation for surgical treatment. We employed inverse probability weighting (IPW) with propensity scores to estimate the average treatment effect (ATE) of race on surgical recommendation. Covariates included age group, tumor grade, income quartile, marital status, metro residence, and histologic subtype. The analysis adjusted for SEER registry (state) and year fixed effects to account for geographic and temporal differences. Multiple imputation with chained equations was used to address missing data and improve the precision.

Results: Among 145,304 women diagnosed with ovarian cancer, 11% were Black, and 89% were White. Compared to White women, Black women were more likely to be younger, unmarried, and reside in large metropolitan areas. Overall, 77.4% of patients received a surgical recommendation, with lower rates among Black women (69.5%) than White women (78.5%). After adjustment for covariates and imputation, IPW analysis revealed that Black women had a 6.8% lower probability of receiving a surgical recommendation compared to White women (ATE = -0.068; 95% CI: -0.080 to -0.057; p < 0.001).

Conclusion: Black women with ovarian cancer are significantly less likely to receive a recommendation for curative surgery than White women, even after adjusting for clinical and sociodemographic factors. These findings underscore the need for systemic interventions to promote equity in oncologic care.

## Introduction

Ovarian cancer is the second most common gynecologic malignancy and the leading cause of gynecologic cancer-related mortality in the United States. It ranks fifth among cancer-related deaths in women [1-3]. According to the National Cancer Institute, an estimated 20,890 new cases and 12,730 deaths are projected for 2025, with an overall five-year survival rate of 51.6%, which declines substantially in advanced-stage disease. The median age at diagnosis of ovarian cancer is 63 years [4-6]. The disease is classified into three primary histological subtypes: epithelial, germ cell, and sex-cord stromal ovarian cancers [7]. Approximately 90% of ovarian cancers are epithelial, of which 70-80% are serous carcinomas diagnosed at stage III [6,8,9]. The etiology of ovarian cancer is multifactorial, involving reproductive, hormonal, genetic, and environmental influences. Many cases are believed to originate from precursor lesions in the fallopian tubes or endometrioid tissue [7]. Established risk factors include advanced age, family history of breast or ovarian cancer, endometriosis, nulliparity, and polycystic ovary syndrome. Modifiable risks such as obesity, smoking, and diet also play a role. Conversely, protective factors include multiparity, oral contraceptive use, physical activity, and tubal ligation [6,7].

Despite advancements in cancer treatment leading to an overall decline in ovarian cancer incidence and mortality, disparities persist across racial, ethnic, and socioeconomic groups [5,8,10]. While African American women have a lower incidence of ovarian cancer than White women, they experience disproportionately higher mortality rates and poorer survival outcomes [3,9-12]. As no effective early detection strategy has been shown to reduce ovarian cancer mortality, obtaining a thorough history remains essential for risk stratification [13]. Diagnosis is further complicated because approximately 95% of patients present with nonspecific symptoms, such as abdominal pain and bloating. About 80% have advanced-stage disease (stage III-IV), with Black women more often presenting later [6]. Other contributing factors to this disparity include more aggressive tumor subtypes, advanced stage at diagnosis, greater comorbidities, limited healthcare access, socioeconomic disadvantages, barriers to genomic testing and emerging therapies, urban racial residential segregation, suboptimal treatment adherence, and a lower likelihood of receiving guideline-concordant care [2,11,14]. Racial and ethnic disparities in ovarian cancer care are closely linked to social determinants of health (SDOH), including socioeconomic status, education, and healthcare access. These disparities contribute to delayed diagnoses, reduced likelihood of undergoing ovarian cancer-specific surgical procedures, lower rates of treatment by high-volume surgeons, and decreased participation in clinical trials that may help elucidate racial and genetic factors influencing treatment outcomes across populations [2].

The economic burden of ovarian cancer is substantial, with treatment costs exacerbated by disparities in healthcare access and quality. Estimated annual care costs in the United States exceed $200,000 during the initial treatment phase, range between $26,000 and $88,000 during continued care, and surpass $129,000 in end-of-life care [15]. On average, African American and Hispanic women face higher out-of-pocket expenses due to lower insurance coverage rates and increased reliance on Medicaid and Medicare, which are associated with reduced access to recommended treatments [16].

Current National Comprehensive Cancer Network (NCCN) guidelines recommend primary tumor cytoreduction or debulking surgery, followed by systemic platinum-based chemotherapy, based on disease stage and patient tolerance [2,15,17]. Although evidence supports the combination of surgery and chemotherapy for optimal outcomes, racial disparities persist, with Black women less likely to receive complete guideline-recommended care compared with White women and less likely to be operated on by high-volume surgeons [2,12]. Addressing these disparities is critical to improving survival outcomes and ensuring equitable care for all patients with ovarian cancer. Thus, this study aims to examine inequities in surgical recommendations for Black and White women with ovarian cancer in the United States.

## Materials and methods

Study design and data source

This retrospective cohort study utilized data from the National Cancer Institute’s Surveillance, Epidemiology, and End Results (SEER) program, which provides comprehensive, population-based information on cancer incidence, treatment, and outcomes in the United States. SEER covers approximately 48% of the U.S. population and is a widely used source for evaluating cancer care disparities [4].

Study population

We included women aged 18 years and older diagnosed with primary ovarian cancer between January 1, 2001, and December 31, 2021. The study focused exclusively on individuals identified as non-Hispanic Black or non-Hispanic White to allow direct comparison between these two racial groups. We excluded cases with missing information on race or surgical recommendation status.

Primary outcome

The primary outcome was the presence of a surgical recommendation for curative treatment, as documented in SEER records. This variable reflects a physician’s decision to recommend surgery, independent of whether surgery was ultimately performed, and serves as a proxy for treatment access and guideline-concordant care.

Independent variable of interest

The primary independent variable was patient race, categorized as non-Hispanic Black versus non-Hispanic White. Race was self-reported and recorded in the SEER database.

Covariates

We adjusted for demographic, clinical, and socioeconomic covariates, including age group at diagnosis, tumor grade, histologic subtype, marital status, income quartile, and metropolitan residence status. To account for geographic and temporal differences, all models included the SEER registry (state) and year fixed effects.

Multiple imputation for missing data

To address missingness in covariates and reduce bias, we employed multiple imputation with chained equations (MICE), generating 20 imputed datasets. This approach assumes that data are missing at random (MAR) conditional on observed variables [19]. Imputation improves estimate precision by preserving the sample size and maintaining statistical power. Results from the imputed datasets were combined using Rubin’s rules to produce final estimates.

Inverse probability weighting (IPW)

We used inverse probability weighting with propensity scores to estimate the average treatment effect (ATE) of race on the likelihood of receiving a surgical recommendation. Propensity scores were calculated using logistic regression, modeling the probability of being Black versus White based on all measured covariates. Stabilized weights were applied to create a pseudo-population in which covariate distributions were balanced across racial groups, reducing confounding bias. This method allows for a more accurate estimation of the causal relationship between race and surgical recommendation in an observational setting [20].

Ethical considerations

This study was a retrospective secondary analysis of publicly available, de-identified data from the Surveillance, Epidemiology, and End Results (SEER) program. Because the data contain no direct patient identifiers and involve no interaction with human subjects, the study was deemed not to constitute human-subject research under U.S. federal regulations. Accordingly, Institutional Review Board approval and informed consent were not required.

Statistical analysis

Weighted logistic regression models were fitted to estimate the effect of race on surgical recommendation, incorporating both IPW weights and imputed datasets. Effect estimates were reported as average treatment effects (ATE) with corresponding 95% confidence intervals (CIs) and p-values. Statistical significance was set at a two-tailed alpha level of 0.05. All analyses were conducted using Stata version 17.

## Results

Among 145,304 women diagnosed with ovarian cancer, 11.1% (n=16,131) were Black and 88.9% (n=129,173) were White (Table 1). Significant differences were observed in sociodemographic and clinical characteristics between the two groups (all p<0.001). Black women were more frequently younger (18-44 years: 14.7% vs. 8.9%), resided in large metropolitan areas (74.5% vs. 59.3%), and were single (36.2% vs. 16.1%) compared with White women. They were also more likely to live in the lowest income quartile (15.9% vs. 9.2%).

**Table 1:** Baseline demographic, clinical, and socioeconomic characteristics of black and white women diagnosed with ovarian cancer in the United States, SEER 2001–2021

Variable	Total Population (N=145,304)	Black (n=16,131)	White (n=129,173)	chi2	p-value
Age				757.90	<0.001
18 - 44 yrs.	13,978 (9.6%)	2,370 (14.7%)	11,608 (8.9%)		
45 - 64 yrs.	58,265 (40.1%)	6,946 (43.1%)	51,319 (39.7%)		
≥ 65 yrs.	73,061 (50.3%)	6,815 (42.3%)	66,246 (51.3%)		
Metro				1.50E+03	< 0.001
Rural areas not adjacent to Metro	6,609 (4.6%)	284 (1.8%)	6,325 (4.9%)		
Rural Areas Adjacent to Metro	12,527 (8.6%)	870 (5.4%)	11,657 (9.0%)		
Small metropolitan	11,877 (8.2%)	964 (5.9%)	10,913 (8.5%)		
Medium Metro	25,608 (17.6%)	1,993 (12.4%)	23,615 (18.3%)		
Large Metro	88,671 (61.0%)	12,018 (74.5%)	76,653 (59.3%)		
Missing	12 (0.01%)	2 (0.01%)	10 (0.01%)		
Marital Status				4.00E+03	<0.001
Single	22,320 (18.3%)	4,725 (36.2%)	17,595 (16.1%)		
Married	57,039 (46.7%)	3,539 (27.1%)	53,500 (49.1%)		
Widowed	24,807 (20.3%)	2,476 (18.9%)	22,331 (20.5%)		
Divorced	12,231 (10.0%)	1,462 (11.2%)	10,769 (9.9%)		
Separated	1,108 (0.9%)	262 (2.0%)	846 (0.8%)		
Unknown	4,586 (3.8%)	602 (4.6%)	3,984 (3.7%)		
Grade				85.13	<0.001
Localized	17,804 (15.2%)	2,065 (15.8%)	15,738 (15.2%)		
Regionalized	20,908 (17.9%)	2,109 (15.6%)	18,799 (18.2%)		
Distant	67,976 (58.2%)	7,940 (58.6%)	60,036 (58.1%)		
Unknown	10,199 (8.7%)	1,375 (10.1%)	8,824 (8.5%)		
Income				1.20E+03	<0.001
Quartile I (4K - 54K)	14,488 (9.9%)	2,565 (15.9%)	11,923 (9.2%)		
Quartile II (55K - 69K)	38,985 (26.8%)	4,562 (28.3%)	34,423 (26.7%)		
Quartile III (70K - 94K)	60,363 (41.6%)	6,837 (42.4%)	53,526 (41.4%)		
Quartile IV (95K -120K)	31,456 (21.7%)	2,165 (13.4%)	29,291 (22.7%)		
Recommend (Surgery)				463.06	<0.001
Not Recommended	22,112 (22.6%)	3,504 (30.5%)	18,608 (21.6%)		
Recommended	75,714 (77.4%)	7,988 (69.5%)	67,726 (78.5%)		
Histology				2.10E+03	<0.001
Sex Cord-Stromal Tumors	2,681 (2.6%)	890 (8.2%)	1,791 (1.9%)		
Epithelial Tumors	89,011 (85.9%)	8,168 (75.6%)	80,843 (87.1%)		
Germ Cell Tumors	1,668 (1.6%)	407 (3.8%)	1,261 (1.4%)		
Small Cell and Other Tumors	10,301 (9.9%)	1,347 (12.5%)	8,954 (9.6%)		

Tumor stage distribution varied, with similar proportions of distant disease in both groups (58.6% vs. 58.1%), but Black women had higher proportions of sex cord-stromal tumors (8.2% vs. 1.9%) and germ cell tumors (3.8% vs. 1.4%), whereas epithelial tumors were more common among White women (87.1% vs. 75.6%) (Table 1).

Overall, 77.4% of patients received a surgical recommendation, but the rate was significantly lower among Black women than White women (69.5% vs. 78.5%) (Table 1).

State-level distribution of ovarian cancer cases by race in the United States

Geographic distribution differed significantly between Black and White women with ovarian cancer (χ² = 6,100, p < 0.001) (Table 2), likely reflecting regional differences in population demographics across states. California had the largest share of cases overall (23.3%), driven largely by White women (24.2% vs. 16.3% for Black women). In contrast, Black women were disproportionately represented in Georgia (18.6% vs. 7.3% for White women) and Louisiana (8.9% vs. 3.2%). New York also showed higher representation among Black women (22.8% vs. 18.7%). By comparison, states such as Idaho, New Mexico, Iowa, and Hawaii had minimal representation of Black women (<0.5%). These patterns reveal clear geographic clustering, with Black women concentrated in southern states and urban centers, while White women were more widely distributed across the United States (Table 2).

**Table 2:** State-level distribution of ovarian cancer cases by race in the United States, SEER 2001-2021

State	Total Population (N=145,304)	Black (n=16,131)	White (n=129,173)	chi2	p-value
				6.10E+03	<0.001
California	33,859 (23.3%)	2,629 (16.3%)	31,230 (24.2%)		
Connecticut	5,453 (3.8%)	332 (2.1%)	3.9%)		
Georgia	12,366 (8.5%)	3,006 (18.6%)	9,360 (7.3%)		
Hawaii	480 (0.3%)	15 (0.1%)	465 (0.4%)		
Idaho	2,053 (1.4%)	3 (0.02%)	2,050 (1.6%)		
Iowa	4,854 (3.3%)	54 (0.3%)	4,800 (3.7%)		
Kentucky	6,006 (4.1%)	272 (1.7%)	5,734 (4.4%)		
Louisiana	5,594 (3.9%)	1,438 (8.9%)	4,156 (3.2%)		
New Jersey	13,301 (9.2%)	1,466 (9.1%)	11,835 (9.2%)		
New Mexico	1,678 (1.2%)	29 (0.2%)	1,649 (1.3%)		
New York	27,855 (19.2%)	3,672 (22.8%)	24,183 (18.7%)		
Seattle	6, 261 (4.3%)	164 (1.0%)	6,097 (4.7%)		
Texas	22,892 (15.8%)	3,041 (18.9%)	19,851 (15.4%)		
Utah	2,652 (1.8%)	10 (0.1%)	2,642 (2.1%)		

Yearly trends in ovarian cancer incidence by race in the United States, 2000-2021

The temporal distribution of ovarian cancer diagnoses from 2000 to 2021 varied significantly between Black and White women (χ² = 481.8, p < 0.001) (Table 3). Early in the study period (2000-2006), the proportion of Black women diagnosed each year ranged from 3.8% to 4.2%, consistently lower than that of White women. From 2010 onward, the proportion of Black women began to rise, surpassing 5% in several later years, including 2014 (5.1%), 2016 (5.2%), and peaking in 2021 (5.6%). In contrast, the proportion of White women showed a gradual decline over time, from 5.1% in 2001 to 3.9% in 2021. These trends suggest a relative increase in the share of diagnoses among Black women over the two-decade period, accompanied by a modest decline among White women (Table 3). 

**Table 3:** Yearly trends in ovarian cancer incidence by race in the United States, SEER 2000–2021

Year	Total Population (N=145,304)	Black (n=16,131)	White (n=129,173)	chi2	p-value
				481.79	<0.001
2000	7,115 (4.9%)	613 (3.8%)	6,502 (5.0%)		
2001	7,255 (4.9%)	615 (3.8%)	6640 (5.1%)		
2002	7,064 (4.9%)	662 (4.1%)	6,402 (4.9%)		
2003	6,981 (4.8%)	679 (4.21%)	6,302 (4.9%)		
2004	6,876 (4.7%)	672 (4.2%)	6,204 (4.8%)		
2005	6,855 (4.7%)	660 (4.1%)	6,195 (4.8%)		
2006	6,989 (4.8%)	637 (3.9%)	6,352 (4.9%)		
2007	6,929 (4.8%)	682 (4.2%)	6,247 (4.8%)		
2008	6,940 (4.8%)	720 (4.5%)	6,220 (4.8%)		
2009	6,848 (4.7%)	723 (4.5%)	6,125 (4.7%)		
2010	6,731 (4.6%)	699 (4.3%)	6,032 (4.7%)		
2011	6,594 (4.5%)	742 (4.6%)	5, 852 (4.5%)		
2012	6,560 (4.5%)	780 (4.8%)	5,780 (4.5%)		
2013	6,594 (4.5%)	776 (4.8%)	5,818 (4.5%)		
2014	6,597 (4.5%)	818 (5.1%)	5,779 (4.5%)		
2015	6,679 (4.6%)	776 (4.8%)	5,903 (4.6%)		
2016	6,318 (4.4%)	838 (5.2%)	5,480 (4.2%)		
2017	6,134 (4.2%)	804 (4.9%)	5,330 (4.1%)		
2018	5,929 (4.1%)	791 (4.9%)	5,138 (3.9%)		
2019	5,854 (4.0%)	787 (4.9%)	5,067 (3.9%)		
2020	5,511 (3.8%)	751 (4.7%)	4,760 (3.7%)		
2021	5,951 (4.1%)	906 (5.6%)	5,045 (3.9%)		

Racial disparities in surgical recommendations

Among women diagnosed with ovarian cancer between 2001 and 2021, inverse probability weighting analysis revealed significant racial disparities in surgical recommendations (Table 4). After adjustment for age, tumor grade, income quartile, marital status, metropolitan residence, histologic subtype, SEER state registry, and year, Black women had a 6.8% lower probability of receiving a surgical recommendation compared with White women (ATE = −0.068; 95% CI, −0.080 to −0.057; p < 0.001). In contrast, the adjusted probability of receiving a surgical recommendation for White women was significantly higher (ATE = 0.784; 95% CI, 0.782 to 0.787; p < 0.001). These differences persisted after multiple imputation to address missing data, underscoring a consistent and clinically meaningful disparity in treatment recommendations by race (Table 4).

**Table 4:** Average treatment effects of race on surgical recommendation for ovarian cancer Average treatment effects (ATE) of Black versus White people on the probability of receiving a surgical recommendation among women with ovarian cancer. Estimates were derived using inverse probability weighting with propensity scores, adjusting for age, tumor grade, income quartile, marital status, metropolitan residence, histologic subtype, states, and year-fixed effects. Multiple imputation addressed missing data. Negative coefficients indicate a lower probability of recommendation compared with White women.

Outcome	Black vs. White	Coef.	Std. Err.	z-value	p-value	95% CI	
Surgical Recommendation	Black	-0.068	0.006	-11.72	<0.001	-0.080	-0.057
	White	0.784	0.001	625.21	<0.001	0.782	0.787

Stage-stratified racial differences in surgical recommendation

Using inverse-probability weighting to adjust for measured confounders and treating White women as the reference, Black women were significantly less likely to receive a surgical recommendation at more advanced stages of ovarian cancer (Table 5). Among women with regional disease, the average marginal effect (risk difference) for Black vs. White was −4.8 percentage points (95% CI, −7.2 to −2.3; p<0.001). The disparity was greatest for distant disease (−7.1 percentage points; 95% CI, −8.9 to −5.3; p<0.001). In contrast, for localized disease, the difference was small and not statistically significant (−0.3 percentage points; 95% CI, −1.2 to 0.7; p=0.564), and for unknown stage, it was −3.4 percentage points (95% CI, −9.2 to 2.5; p=0.260). Collectively, these estimates indicate that racial disparities in surgical recommendations widen with increasing stage at presentation, with no detectable difference at the localized stage (Table 5).

**Table 5:** Stage-stratified racial differences in surgical recommendation among women with ovarian cancer (Black vs. White) Entries represent average marginal effects (dy/dx) as absolute percentage-point differences in surgical recommendation probabilities for Black versus White women, with White as the reference group. Negative values indicate lower rates for Black women. Estimates were derived using inverse-probability weighting, adjusting for age, marital status, location, income, tumor histology, and fixed effects for state and year. 95% CIs and two-sided p-values are shown. Results are stratified by cancer stage (localized, regionalized, distant, unknown).

Black (ref. White)	dy/dx	Std. Err.	z	95% CI		P>z
Grade						
Localized	-0.003	0.005	-0.580	-0.012	0.007	0.564
Regionalized	-0.048	0.012	-3.820	-0.072	-0.023	<0.001
Distant	-0.071	0.009	-7.790	-0.089	-0.053	<0.001
Unknown	-0.034	0.030	-1.130	-0.092	0.025	0.260

Histology-stratified racial differences in surgical recommendations

Using inverse-probability weighting and treating White women as the reference, the Black race was associated with a lower likelihood of receiving a surgical recommendation in several histologic subtypes (Table 6). The disparity was largest for small cell and other rare tumors (average marginal effect, AME = −8.4 percentage points; 95% CI, −13.4 to −3.5; p=0.001) and was also significant for epithelial tumors (AME = −5.3 percentage points; 95% CI, −6.6 to −4.0; p<0.001). For germ cell tumors, the estimated difference was −4.5 percentage points (95% CI, −10.6 to 1.7; p=0.156), and for sex cord-stromal tumors, it was +2.4 percentage points (95% CI, −0.4 to 5.1; p=0.098); neither reached statistical significance. Overall, these findings indicate that racial disparities in surgical recommendations are most pronounced in epithelial and rare histology, with no detectable difference in sex cord-stromal or germ cell tumors (Table 6).

**Table 6:** Histology-stratified racial differences in surgical recommendation among women with ovarian cancer (Black vs. White) Entries represent the average marginal effects (dy/dx) showing the percentage-point differences in the probability of receiving a surgical recommendation for Black versus White women (with White as the reference group). Negative values indicate lower rates for Black women. The estimates were derived using an inverse-probability weighting framework, adjusting for variables such as age, marital status, metropolitan residence, neighborhood income, and tumor grade, while controlling for state and year fixed effects. Results are stratified by histologic subtype, and p-values and 95% confidence intervals are provided.

Black (ref. White)	dy/dx	Std. Err.	z	95% CI		P>z
Histology						
Sex Cord-Stromal Tumors	0.024	0.014	1.660	-0.004	0.051	0.098
Epithelial Tumors	-0.053	0.007	-8.080	-0.066	-0.040	<0.001
Germ Cell Tumors	-0.045	0.031	-1.420	-0.106	0.017	0.156
Small Cell and Other Rare Tumors	-0.084	0.025	-3.360	-0.134	-0.035	0.001

Stage- and histology-specific racial disparities in surgical recommendations for ovarian cancer

**Figure 1:**
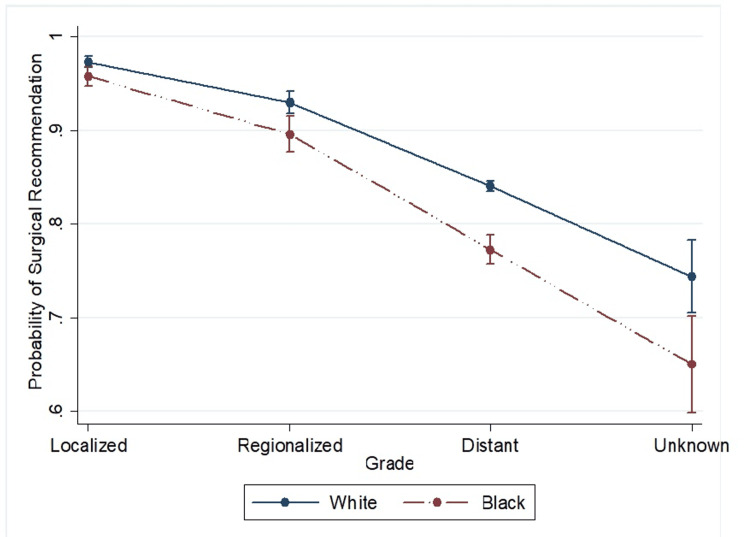
Probability of surgical recommendation by stage Stage-stratified probability of receiving a surgical recommendation among Black and White women with ovarian cancer in the United States, 2001–2021. Estimates were derived using inverse-probability weighting adjusted for demographic, clinical, and socioeconomic covariates, with White women as the reference group. Racial disparities were minimal at localized stages but widened substantially at regional and distant stages, with Black women significantly less likely to receive a recommendation for surgery. Error bars indicate 95% confidence intervals.

**Figure 2:**
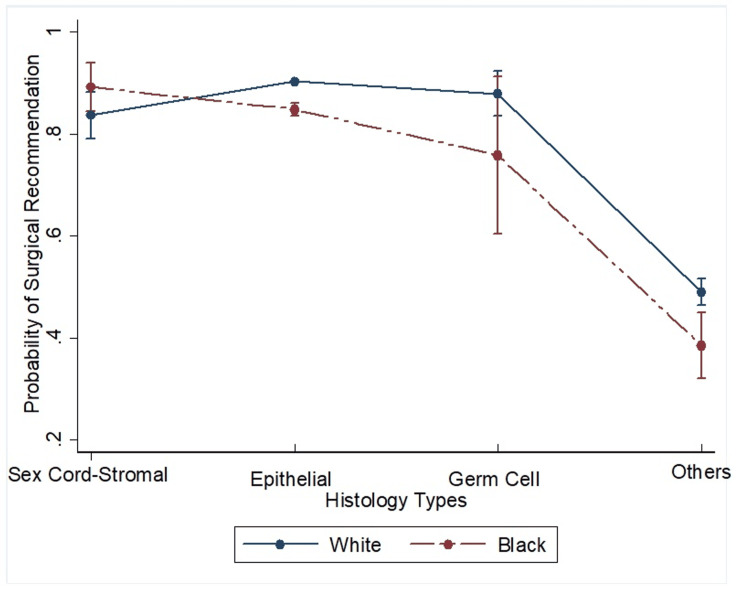
Probability of surgical recommendation by histologic subtypes Histology-stratified probability of receiving a surgical recommendation among Black and White women with ovarian cancer in the United States, 2001–2021. Inverse-probability weighted models adjusted for relevant covariates revealed significant racial disparities for epithelial tumors and rare histologies, with Black women less likely to receive surgical recommendations compared with White women. No significant differences were observed for sex cord–stromal or germ cell tumors. Error bars indicate 95% confidence intervals.

Figures 1, 2 highlight pronounced racial disparities in surgical recommendations for ovarian cancer across stage and histologic subtype. Using inverse-probability weighting to adjust for demographic, clinical, and socioeconomic factors, the probability of a surgical recommendation was high and similar for White and Black women at localized disease, but racial differences widened with advancing stage: at the regional stage, White women had a probability near 0.9 versus about 0.8 for Black women, and at the distant stage, the differential widened further, with probabilities falling to roughly 0.7 for Black women (Figure 1). When stratified by histologic subtype, disparities were most evident for epithelial and other rare tumor types (Figure 2); Black women were significantly less likely than White women to receive surgical recommendations for these cancers, whereas no meaningful differences were seen for sex cord-stromal or germ cell tumors. These findings suggest that both disease stage and histology modify racial inequities in the surgical management of ovarian cancer.

Cancer-specific survival by race and surgical recommendation

**Figure 3:**
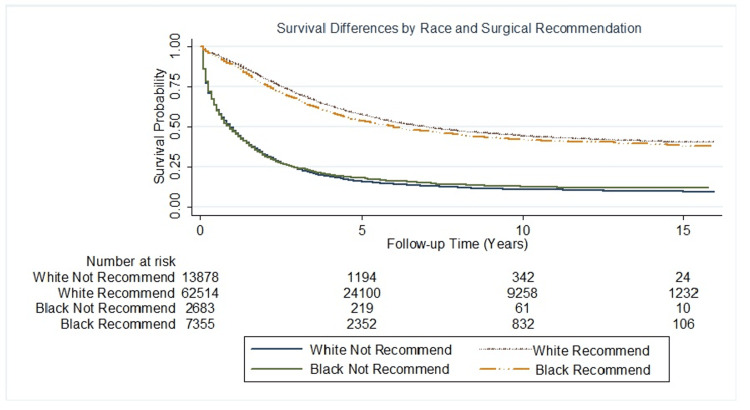
Cancer-specific survival by race and surgical recommendation Kaplan–Meier curves for cancer-specific survival indicate that both Black and White women with ovarian cancer had higher survival probabilities when they received a surgical recommendation. This survival advantage persisted over a 15-year follow-up period. Although Black women with a surgical recommendation had better outcomes than those without, their survival rates remained lower than those of White women, even after surgery was recommended. Numbers at risk at baseline and selected time points are provided below the curves.

Kaplan-Meier analysis demonstrated marked differences in cancer-specific survival according to both race and receipt of a surgical recommendation (Figure 3). Across all racial groups, patients who received a surgical recommendation had substantially higher survival probabilities throughout follow-up compared with those without a recommendation. The survival benefit associated with a surgical recommendation was evident early and persisted over the 15-year follow-up period.

Among White women, those who received a surgical recommendation had the most favorable survival, while White women without a recommendation experienced markedly lower survival. Similarly, Black women who were recommended surgery had significantly better outcomes than those without a recommendation. However, even with a surgical recommendation, survival among Black women was lower than that of their White counterparts, suggesting that racial disparities extend beyond treatment recommendations to include differences in treatment receipt, quality of care, baseline health status, and socioeconomic conditions (Figure 3).

## Discussion

In this comprehensive, population-based study of more than 145,000 women diagnosed with ovarian cancer in the United States between 2001 and 2021, we observed striking racial inequities in surgical treatment recommendations. Black women were consistently and significantly less likely than their White counterparts to receive a recommendation for potentially curative surgery, even after rigorous adjustment for clinical, sociodemographic, and geographic factors through inverse probability weighting. Importantly, this disparity endured across nearly all tumor grades and histologic subtypes, and it coincided with worse cancer-specific survival among Black women. Despite the rising proportion of ovarian cancer diagnoses among Black women in recent years, equitable surgical recommendations have not been achieved. Our Kaplan-Meier survival analyses further affirmed the pivotal role of surgical recommendation, demonstrating that patients who were advised to undergo surgery experienced markedly improved survival outcomes. These findings highlight not only the survival benefit of surgical intervention but also the profound consequences of inequitable treatment recommendations on population-level outcomes.

Potential explanations for racial disparities

Several factors may contribute to the observed differences in surgical recommendations. Implicit provider bias and structural racism within healthcare systems have been documented as contributors to disparities in oncologic treatment [17,21]. Black women may present to hospitals with fewer gynecologic oncology resources, be more frequently treated in under-resourced facilities, or experience delays in diagnosis. In some cases, care delivered by non-gynecologic oncologists, such as general gynecologists, may contribute to lower rates of guideline-concordant surgical management [17,22]. Additionally, differences in patient-provider communication, cultural mistrust stemming from historical injustices, and lower referral rates from primary care may influence treatment pathways [17,23]. Structural factors within the health insurance system, including differences in insurance coverage, reimbursement policies, and reliance on public insurance programs, may further limit access to specialized oncologic care. These insurance-related barriers, along with socioeconomic constraints, such as reduced healthcare affordability and availability, may intersect with race to exacerbate disparities [3,16,22,24].

Comparison with prior literature

Our findings align with prior research demonstrating lower rates of ovarian cancer surgery among Black women compared to White women. Studies have shown that even when adjusting for stage and comorbidity, Black women are less likely to receive guideline-concordant surgical care and are more often treated by non-specialists [17,22]. Unlike many prior studies, our use of IPW with multiple imputation strengthens causal inference by accounting for a wide range of confounders and mitigating bias from missing data.

Policy implications

These findings have important policy implications, underscoring the need to make equitable access to gynecologic oncology services a national priority. Efforts should focus on implementing standardized surgical referral protocols across all hospitals treating ovarian cancer, expanding referral networks to ensure that patients from underserved areas are connected to high-volume cancer centers, and enhancing cultural competence alongside implicit bias training for oncology care teams. Additionally, strengthening patient navigation programs can provide targeted support to Black women throughout the diagnostic and treatment processes [23,25]. Given the strong survival benefit associated with surgical recommendations, interventions that address disparities at the referral stage have the potential to improve overall survival and advance health equity meaningfully.

Limitations

This study has several limitations. The SEER registry does not capture important clinical and socioeconomic variables, including comorbidity profiles, performance status, patient preferences, and insurance status, all of which may influence treatment recommendations and outcomes. The absence of insurance information is particularly relevant, as coverage status is a key determinant of access to care and may confound observed associations. Additionally, we were unable to account for other potential unmeasured confounders due to data limitations. Provider-level factors, such as clinician decision-making, and institutional characteristics, including resource availability, were also not captured and may contribute to observed disparities.

Prospective studies incorporating more granular clinical, socioeconomic, and provider-level data are needed to better elucidate the underlying drivers of lower surgical recommendation rates among Black women with ovarian cancer. Nevertheless, the large sample size, national scope, and rigorous statistical methodology strengthen the robustness of our findings.

## Conclusions

Black women with ovarian cancer are significantly less likely to receive a recommendation for curative surgery than White women, a disparity that remains after adjustment for key clinical and sociodemographic factors. Surgical recommendation is a pivotal determinant of survival, and inequities at this stage may contribute to poorer outcomes among Black women. Future research should incorporate linked administrative claims to evaluate the role of insurance and comorbidities and assess interventions, such as policy mandates, care navigation programs, and regionalization of care, that can eliminate these disparities.
